# Mitochondrial Dysfunction Causes Oxidative Stress and Tapetal Apoptosis in Chemical Hybridization Reagent-Induced Male Sterility in Wheat

**DOI:** 10.3389/fpls.2017.02217

**Published:** 2018-01-10

**Authors:** Shuping Wang, Yingxin Zhang, Qilu Song, Zhengwu Fang, Zheng Chen, Yamin Zhang, Lili Zhang, Lin Zhang, Na Niu, Shoucai Ma, Junwei Wang, Yaqin Yao, Zanmin Hu, Gaisheng Zhang

**Affiliations:** ^1^Key Laboratory of Crop Heterosis of Shaanxi Province, College of Agronomy, Northwest A&F University, National Yangling Agricultural Biotechnology and Breeding Center, Yangling Branch of State Wheat Improvement Centre, Wheat Breeding Engineering Research Center, Ministry of Education, Yangling, China; ^2^Hubei Key Laboratory of Waterlogging Disaster and Agricultural Use of Wetland, College of Agronomy, Yangtze University, Jingzhou, China; ^3^Institute of Genetics and Developmental Biology, Chinese Academy of Sciences, Beijing, China; ^4^Department of Anaesthesia and Intensive Care, The Chinese University of Hong Kong, Hong Kong, China; ^5^College of Life Sciences, Northwest A&F University, Yangling, China

**Keywords:** mitochondrial dysfunction, oxidative stress, tapetal programmed cell death, chemical hybridization agent, wheat

## Abstract

Male sterility in plants has been strongly linked to mitochondrial dysfunction. Chemical hybridization agent (CHA)-induced male sterility is an important tool in crop heterosis. Therefore, it is important to better understand the relationship between mitochondria and CHA-induced male sterility in wheat. This study reports on the impairment of mitochondrial function duo to CHA-SQ-1, which occurs by decreasing cytochrome oxidase and adenosine triphosphate synthase protein levels and theirs activities, respiratory rate, and in turn results in the inhibition of the mitochondrial electron transport chain (ETC), excessive production of reactive oxygen species (ROS) and disruption of the alternative oxidase pathway. Subsequently, excessive ROS combined with MnSOD defects results in damage to the mitochondrial membrane, followed by ROS release into the cytoplasm. The microspores underwent severe oxidative stress during pollen development. Furthermore, chronic oxidative stress, together with the overexpression of type II metacaspase, triggered premature tapetal apoptosis, which resulted in pollen abortion. Accordingly, we propose a metabolic pathway for mitochondrial-mediated male sterility in wheat, which provides information on the molecular events underlying CHA-SQ-1-induced abortion of anthers and may serve as an additional guide to the practical application of hybrid breeding.

## Introduction

Heterosis plays a major role in improving crop yields, and has been used in crop production for decades. Its role in wheat (*Triticum aestivum* L.) breeding is underdeveloped despite its early analysis by Freeman (1919), after which the possibility of commercial exploitation of heterosis in wheat was suggested (Briggle, 1963; Zhang et al., 2013). However, as wheat a typically self-pollinating crop, using chemical hybridizing agent (CHA) to induce male sterility because female parentals generally do not undergo self-pollination are crucial for the commercial production of hybrid wheat seeds. Recently, more than applicable 40 CHAs have been reported (Singh et al., 2015b), some of which are found effective in inducing pollen sterility, such as RH007 (Mizelle et al., 1989), SC2053 (Fan et al., 1998), and Genesis (Singh et al., 2010). Compared with them, SQ-1 is an ideal chemical hybridization agent which could induce male sterility by changing the cell microstructure (Wang et al., 2016), triggering programmed cell death (PCD) (Wang et al., 2015a, 2016), striking the oxidative/antioxidative balance (Wang et al., 2016) and increasing the cell membrane permeability (Song et al., 2015). Despite some progress toward understanding the mechanisms of male sterility has been made, such as reactive oxygen and aliphatic metabolism (Ba et al., 2013, 2014a), DNA methylation (Ba et al., 2014b), cell morphological (Wang et al., 2015a), transcriptome (Zhu et al., 2015) and proteomics (Song et al., 2015; Wang et al., 2015b), the mechanisms underlying male sterility in plants resulting from CHA treatment remain elusive.

Mitochondria in higher plants are the main source of adenosine triphosphate (ATP) formation, providing chemical energy for plant development, productivity, fertility and resistance to disease (Chen et al., 2010; Tang et al., 2014). Previous studies on the mitochondrial genome have indicated that mutation and recombination have a direct relationship with CMS (cytoplasmic male-sterility) and is therefore suggestive of the role of the mitochondrial genome as a carrier of genes involved in fertility (Linke and Borner, 2005; Wang K. et al., 2013). Some genes and/or open reading frames (ORFs) are often chimeric and co-transcribed with genes that encode the mitochondrial subunits of the ETC enzymes (complexes I–IV) or ATP synthase (complex V) or fragmented versions of the functional mitochondrion-based complexes (Hanson and Bentolila, 2004; Linke and Borner, 2005; Yang et al., 2012; Kitazaki et al., 2015). For example, *orf256* is fused and co-transcribed with *coxI* in T-CMS of wheat (Yang et al., 2012), *orf79* with complex III in rice (Wang K. et al., 2013), and *orf222* with *nad5* in CMS of *Brassica nap* (Brown et al., 1998). Meanwhile, variations in mitochondrial ETC (mtECT) complex subunit gene expression might lead to energy deficiency and oxidative stress during anther development, thus triggering pollen abortion (Sabar et al., 2000; Luo et al., 2013). Therefore, more research on mitochondria may help elucidate the mechanism underlying male sterility.

Apoptosis is a genetically determined process that occurs in all multicellular organisms and involves self-activated cell death (Zhao et al., 2015); it is essential for growth and development of multicellular organisms as well as for proper environmental responses (Gadjev et al., 2008), especially for plants surviving in adverse environments such as those involving biotic and abiotic stresses (Suzuki et al., 2012; Zhao et al., 2015). Plant apoptosis is involved in the vegetative and reproductive stages of development, including leaf senescence, floral organ abscission, embryo formation, and pollen self-incompatibility (Lam et al., 2001; Thomas and Franklin-Tong, 2004; Shi et al., 2015). During anther development, tapetal cell degradation and anther dehiscence serve as the main characteristics of apoptosis male sterility (Jung et al., 2005; Vizcay-Barrena and Wilson, 2006; Zhang et al., 2010; Shi et al., 2015). Recently, mitochondria-mediated tapetum apoptosis studies in higher plants have made some progress (Li et al., 2012; Luo et al., 2013). In plants, reactive oxygen species (ROS), which are predominantly generated in the mitochondrial respiratory chain, often disrupts mitochondrial metabolism (Navrot et al., 2007). Furthermore, mtETC complexes I and III are considered as the major sites of ROS production (Chen et al., 2003). Increasing evidence indicates that ROS acts as an important regulator of cell growth, and plays a key role in apoptosis of the tapetum, because its spatial distribution influences anther morphological development (Li et al., 2012; Luo et al., 2013).

These previous studies were mainly based on CMS and pointed to an association between CMS and mitochondria. To better understand the complex mechanisms between mitochondria and CHA-induced male sterility in wheat, a metabolic pathway of anther abortion in CHA-induced male sterility has been established in the present study, which has revealed the strong correlation between mitochondria and CHA-induced male sterility in wheat. The results presented here might help explain the male sterility in wheat that resulted from CHA treatment and provide a reference for further study on gene regulatory mechanisms underlying male sterility involving wheat.

## Materials and Methods

### Plant Material and Treatments

SQ-1, provided by Key Laboratory of Crop Heterosis of Shaanxi Province, is a new pyridazine compound, the main ingredient of which is 4-chloroaniline (Wang et al., 2015a). Details of the wheat treatment were as previously described (Liu et al., 2014; Wang et al., 2015a). Wheat cultivar “Xinong 1376” was treated by SQ-1 at a rate of 5.0 kg/ha, and sprayed when the crop was around the 8.5 stage based on the Feekes’ scale (Large, 1954; Wang et al., 2016). All plants were conventionally grown in a wheat field of the experimental station of the Northwest Agriculture and Forestry University in Yangling, China. The pollen developmental stages were as described elsewhere (Wang et al., 2015a). Florets, with glumes and awns removed (early uninucleate stage and trinucleate stage, respectively), were harvested for mitochondria isolation. Anthers at five stages (tetrad stage, early uninucleate stage, later-uninucleate stage, binucleate stage and trinucleate stage) were also collected. Anther soluble sugar levels were measured using anthrone colorimetry method as described by Oliver et al. (2005), and starch content was measured according to Dorion et al. (1996) and Wang et al. (2016).

### Phenotype Analyses

Images of plant materials were captured with a Nikon E995 digital camera (Nikon, Japan) attached to a Motic K400 dissecting microscope (Preiser Scientific, Louisville, KY, United States). For transmission electron microscopy observation, anthers were fixed, embedded, and then stained followed the procedure of Wang H. et al. (2013), and assessed using a JEM-1230 transmission electron microscope (JEOL, Tokyo, Japan).

### Mitochondrial Protein Analysis

Mitochondria were isolated using the procedure described by Chen et al. (2010). Freshly collected florets were transferred to a precooled Waring blender and incubated in homogenization solution; the homogenate was filtered through four layers of Miracloth (Calbiochem, San Diego, CA, United States), followed by gradient centrifugation. The mitochondria-enriched pellet was resuspended and used for mitochondrial protein isolation. The protein concentration was measured using the Bradford method, as described by Zuo and Lundahl (2000).

2-DE was conducted as described by Song et al. (2015) and the manufacturer’s instructions (Bio-Rad, Hercules, CA, United States). About 160 μg of total mitochondrial protein was separated by loading the sample on a 17-cm (pH 4–7) linear pH gradient IPG strip (Bio-Rad), and subjected to electrophoresis on the IPGphor apparatus (PROTEAN IEF Cell; Bio-Rad, United States) for 80 kV-h. The second electrophoretic dimension was conducted using 11% SDS-PAGE. Protein spots were visualized by silver staining. The 2-DE gels were scanned using a UMAX PowerLook 2100XL scanner (UMAX Systems GmbH, Willich, Germany) and analyzed with PDQuest 2-DE 8.0.1 (Bio-Rad) software. Only spots altered over 1.3-fold (*p* ≤ 0.05) were considered as differentially expressed proteins. Three independent experiments were performed as biological replicates for all experiments. For in-gel digestion, MS analysis and database searching of differentially expressed proteins (DEPs) were carried out as described by Song et al. (2015).

### Cytochrome *c* Oxidase (COX) and ATPase Activities Measurements

COX and ATPase in purified mitochondria were extracted using freeze-thaw cycles in the presence of 0.05% Triton X-100 and the activities of COX and ATPase were determined according to Ji et al. (2013).

### Subcellular Localization of Hydrogen Peroxide

Hydrogen peroxide in anthers was localized at the ultrastructural level by using the cerium chloride method of Luo et al. (2013). Briefly, wheat anthers at various developmental stages were incubated in a fresh solution of 10 mM CeCl_3_ (Sigma–Aldrich, United States) in a 50 mM 3-(*N*-morpholino) propanesulfonic acid (MOPS) buffer (pH 7.2). The washed, fixed and dehydrated anthers were embedded in Eponate resin (Ted Pella Inc., Redding, CA, United States). Ultrathin sections were evaluated without further staining under a transmission electron microscope (JEM-1230, JEOL, Tokyo, Japan).

### Detection and Measurement of ROS

ROS produced in the anthers at different developmental stages was measured using 2′,7′-dichlorodihydrofluorescein diacetate (H_2_DCF-DA; Sigma–Aldrich). Anthers were embedded in an optimal cutting temperature medium (Sakura Finetek, Torrance, CA, United States), frozened, and then sectioned. Sections (10-μm) and fresh microspores were incubated in 5 μM H_2_DCF-DA (Sigma–Aldrich) in DMSO (Sigma–Aldrich). The fluorescent DCF signals were detected by a fluorescence microscope (Olympus BX 51, Olympus, Japan).

O_2_^-^ and H_2_O_2_ contents were calculated using the procedure of Song et al. (2015). Lipid peroxidation was determined by calculating the malondialdehyde (MDA) level as described by Wang et al. (2016).

### Enzyme Assays

Wheat anthers at various developmental stages were collected and used for measurement. Activities of superoxide dismutase (SOD), catalase (CAT) and guaiacol peroxidase (POD) were measured using the procedure of Song et al. (2015). The activity of manganese superoxide dismutase (MnSOD) was determined according to Prochazkova et al. (2001).

### Respiratory Activity Measurements

The individual activities of the cytochrome oxidase pathway (COP; V_cyt_) and alternative oxidase pathway (AOP; V_alt_) in the anthers were calculated by multiplying the total respiration rate (V_t_) and then measured using the method of Vanlerberghe and McLntosh (1996).

### qPCR Assay

All primers used in the analysis are listed in **Supplementary Table S1**. Total RNA was isolated from the anthers at various stages using Trizol Reagent Kit (Invitrogen, Carlsbad, CA, United States) and used for first-strand cDNA synthesis with a PrimeScript^TM^ RT reagent Kit (Takara Bio, Tokyo, Japan), following the manufacturer’s protocol. qPCR analysis was performed with SYBR Premix EX Taq (Takara Bio) on CFX96^TM^ Real-Time PCR Detection System (Bio-Rad) according to the manufacturer’s instructions. All reactions were performed in triplicate on one plate and repeated three times (biological replicates). Gene expression levels were normalized using the *ACTIN* gene, and the relative expression levels were calculated using the 2^-^*^ΔΔ^*^Ct^ analysis method.

### Cell Vitality and Membrane Integrity Assay with FDA

The collected microspores were washed in PBS, followed by immediate suspension in 10 μg ml^-1^ of fluorescein diacetate (FDA; Sigma–Aldrich) for 30 min at 25°C in the dark to maximize formation of fluorescein. Microspores were assessed using fluorescence microscope (for details see detection of ROS).

## Results

### Phenotypic and Histological Analyses of CHA-SQ-1-Induced Male Sterile in Wheat

**FIGURE 1:**
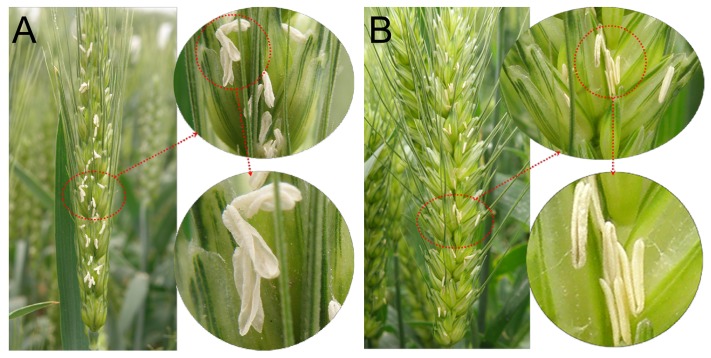
Comparison of spikes after pollen shedding. **(A)** The control of wheat plants. **(B)** The CHA-SQ-1 treatment of wheat plants.

The experimental materials were applied at 5.0 kg ha^-1^ SQ-1 at the Feekes’ 8.5 stage to induce male sterility. In mature plants, even though pistils of control and CHA-SQ-1-treated plants were normally developed and able to generate normal seeds after backcrossing with fertile pollen, the anthers of CHA-SQ-1-treated plants were apparently smaller than those of controls, and had no mature pollen (Wang et al., 2015a). Also, unlike the mature pollen of control plants, those of the CHA-SQ-1-treated plants did not show intense staining with iodine-potassium iodide (Wang et al., 2015a) and the pollen was not shed (**Figure 1**). These results indicated that CHA-SQ-1-treated plants were 100% pollen sterile.

**FIGURE 2:**
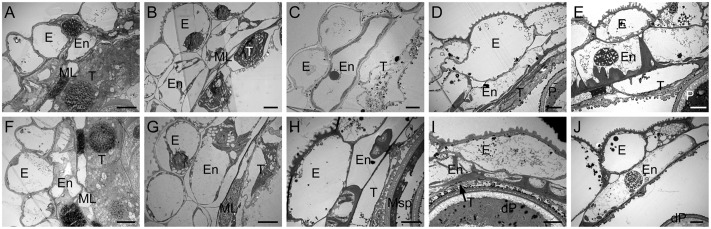
Transmission electron micrographs of the anthers from control **(A–E)** and the CHA-SQ-1 treatment **(F–J)** of wheat plants. **(A,F)** Tetrad stage. **(B,G)** Early uninucleate stage. **(C,H)** Later-uninucleate stage. **(D,I)** Binucleate stage. **(E,J)** Trinucleate stage. E, epidermis; En, endothecium; ML, middle layer; T, tapetum; Msp, microspore; P, pollen; dP, degenerated pollen. Bars = 10 μm.

To determine the morphological alterations of anthers in CHA-SQ-1-treated plants, transverse sections of these germinal organs were assessed (**Figure 2**). Based on the morphological landmarks or cellular stages observed by light microscope and the previously established classification system of anther development (Song et al., 2014), wheat anther development were classified into five stages (early uninucleate stage, later-uninucleate stage, binucleate stage and trinucleate stage). In the tetrad stage, no distinct differences between control and CHA-SQ-1-treated plants were detected, the tapetal cytoplasm presented dense agglomerates, and the middle layer showed a band-like shape. Furthermore, the tapetum and microsporocytes apparently underwent normal development as well as meiosis to produce tetrads of haploid microspores (**Figures 2A,F** and **Supplementary Figures S2A,F**). At the early uninucleate stage, young microspores were released from tetrads of control plants, whereas the cells of tapetum began to degenerate, the middle layers became very thin and remained distinct in both control and CHA-SQ-1-treated plants (**Figures 2B,G** and **Supplementary Figures S2B,G**). Subsequently, from later-uninucleate to trinucleate stage, the microspores of the control plants underwent two rounds of mitosis, thus generating mature trinucleate pollen grains. The tapetum remained visible (**Figures 2C–E** and **Supplementary Figures S2C–E**). In contrast, the tapetum was completely degraded and was invisible in CHA-SQ-1-treated plants at the trinucleate stage (**Figures 2H–J** and **Supplementary Figures S2H–J**). These results show that CHA-SQ-1-treated plants suffered anther immaturity, especially tapetal degeneration and pollen abortion.

### Inhibition of Mitochondrial Electron Transport

**FIGURE 3:**
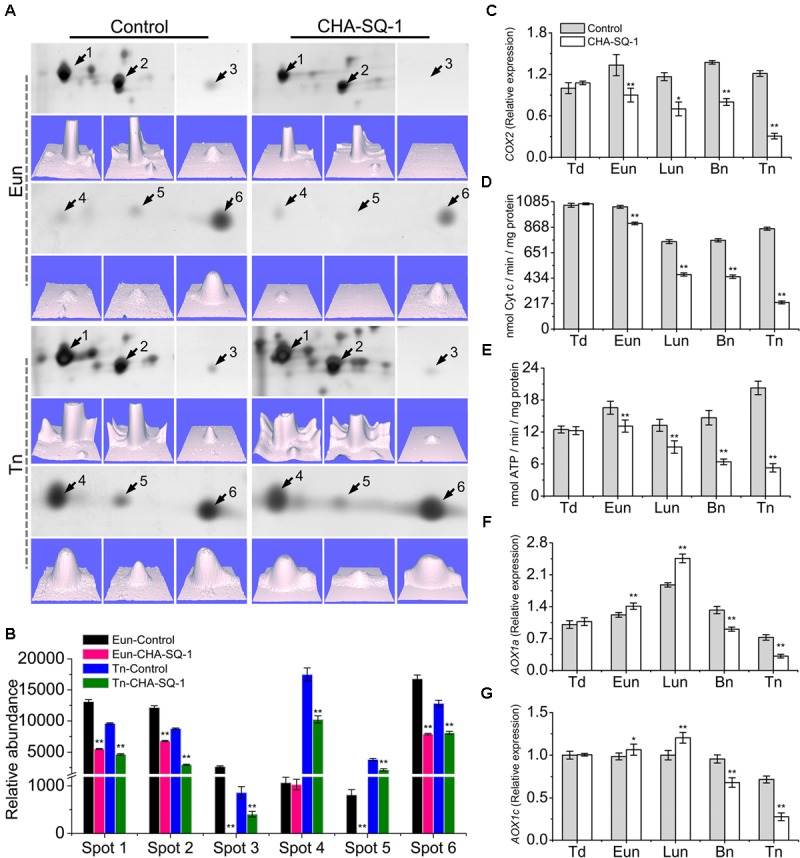
Inhibition of mitochondrial electron transport in the CHA-SQ-1 treatment of wheat plants. **(A)** Six differentially expressed proteins (DEPs) of floret mitochondria at the early-uninucleate (Eun) and trinucleate (Tn) stages, and the corresponding three-dimensional images (*lower panels*) of expression using PDQuest software. **(B)** Abundance profiles of these differentially expressed proteins. Spots 1, 2, atp1; spots 3, 5, 6, putative cytochrome *c* oxidase; spots 4, cytochrome b5; more details are presented in **Supplementary Table S2**. **(C,F,G)** qPCR quantification (2^-^*^ΔΔ^*^Ct^ method) of *COX2* **(C)**, *AOX1a* **(F)**, and *AOX1c* **(G)** in anthers of CHA-SQ-1-treated wheat plants compared with its counterpart stages of control treatment. **(D,E)** Activities of mitochondrial cytochrome *c* oxidase and ATPase in wheat florets at different developmental stages. Td, tetrad stage; Eun, early uninucleate stage; Lun, later-uninucleate stage; Bn, binucleate stage; Tn, trinucleate stage. Data are means ± SD of three independent experiments (biological replicates). The significant of differences was assessed by Student’s *t*-test (^∗^*P* < 0.05, ^∗∗^*P* < 0.01).

Previous studies have demonstrated that the proteomics of mitochondria was altered in CHA-SQ-1-treated plants, especially the greatly reduced expression of mtETC/ATP synthesis-related proteins (Wang et al., 2015b; **Figures 3A,B**, **Supplementary Figure S1**, and **Supplementary Table S2**). At the early uninucleate stage, atp1 (spots 1, 2) and putative cytochrome *c* oxidase subunit (spots 3, 5, 6) were down-regulated over 1.8-fold in the CHA-SQ-1-treated plants. At the trinucleate stage, six DEPs (spots1-6, **Supplementary Table S2**) were significantly down-regulated (>1.5-fold) in CHA-SQ-1-treated plants. These results indicated that mtETC complexes IV (i.e., cytochrome *c* oxidase) and V (i.e., ATP synthase) were significantly down-regulated from early uninucleate stage to trinucleate stage.

To determine the temporal patterns of COX expression more precisely, *COX2*, a subunit of COX, were selected for further analysis (**Figure 3C**). *COX2* proteins were conserved in eukaryotes (**Supplementary Figure S3**) and functioned in the assembly of COX, the variant *COX2* subunit was associated with male sterility (Chase and Gabaylaughnan, 2004). qPCR analysis showed no obvious differences in expression levels at the tetrad stage. However, from early uninucleate to trinucleate stage, the expression of *COX2* in CHA-SQ-1-treated plants was markedly reduced, which was reduced 1.5-fold at the early uninucleate stage, 1.7-fold at the later-uninucleate stage, 1.8-fold at the binucleate stage and 4.0-fold at the trinucleate stage, respectively (**Figure 3C**).

In addition, the activity of COX and ATPase in mitochondria were assessed (**Figures 3D,E**), a significant decrease of COX and ATPase activities in CHA-SQ-1-treated plants was observed from early uninucleate to trinucleate stage.

In the mitochondria of higher plants, electron flow can proceed via the COP and/or AOP. In the above experiments, the COP in the anther of CHA-SQ-1-treated plants was inhibited, thus excess electrons could precede the alternative oxidase (AOX) reducing the level of superoxide to inhibit apoptosis (Fleury et al., 2002). To further understand the dynamic changes of AOP in CHA-SQ-1-treated plants, the expression levels of the *AOX1a* (**Figure 3F**) and *AOX1c* (**Figure 3G**) genes were analyzed using qPCR at the five described stages. The PCR results showed that there was detectable expression of *AOX1a* and *AOX1c* in the anther and no distinct differential expressions at the tetrad stage. However, from the early uninucleate to the later-uninucleate stage, the *AOX1a* and *AOX1c* were strongly expressed in the anthers of CHA-SQ-1-treated plants. In addition, a marked increase in the transcript level of AOX was observed, indicating that the AOP temporarily accepts excess electrons in the anther when the COP was inhibited at the early uninucleate stage. However, starting from the binucleate stage, the *AOX1a* and *AOX1c* showed lower levels and reached the minimum at the trinucleate stage. Thus, the AOP in the anther of CHA-SQ-1-treated plants was also inhibited.

**FIGURE 4:**
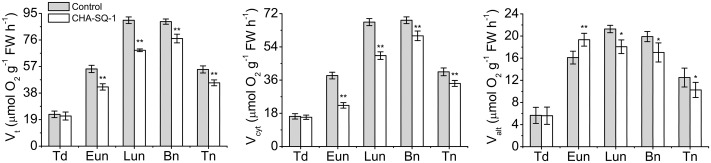
Analysis of respiratory activity. Total respiration (V_t_), and activities of the cytochrome pathway (V_cyt_) and the alternative pathway activity (V_alt_) in control and CHA-SQ-1-treated wheat plants. Eun, early uninucleate stage; Lun, later-uninucleate stage; Bn, binucleate stage; Tn, trinucleate stage. Data are means ± SD of three independent experiments (biological replicates). The significant of differences was assessed by Student’s *t*-test (^∗^*P* < 0.05, ^∗∗^*P* < 0.01).

Taken together, these physiological alterations occur upon disruption of the mitochondrion, particularly that relating to electron transport. Therefore, CHA-SQ-1 impairs the electron transport efficiency, which is followed by a decrease in the rate of ATP production and respiration (**Figure 4**).

### ROS Released from Mitochondria

**FIGURE 5:**
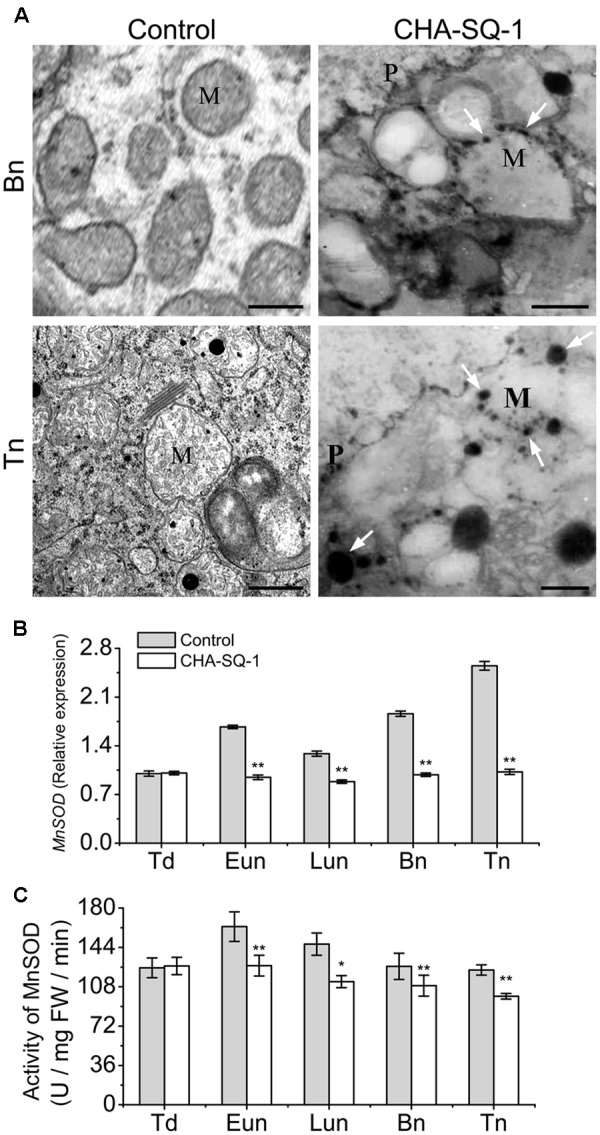
Analysis of mitochondrial ROS production. **(A)** Transmission electron microscopy localization of hydrogen peroxide (H_2_O_2_; black precipitates) in pollen grains. Electron-dense deposits of cerium perhydroxide (shown as black precipitates and indicated by white arrows) depict the accumulation of hydrogen peroxide around the external membranes of the mitochondria of pollen grains of CHA-SQ-1-treated plants at the binucleate stage (Bn) and the trinucleate stage (Tn). M, mitochondrion; P, pollen grain wall. Bars = 0.5 μm. **(B)** qPCR expression of MnSOD in the anthers of CHA-SQ-1-treated wheat plants compared with its counterpart stages of control treatment. **(C)** Activity of MnSOD in anther at different developmental stages. Td, tetrad stage; Eun, early uninucleate stage; Lun, later-uninucleate stage; Bn, binucleate stage; Tn, trinucleate stage. Data are means ± SD of three independent experiments (biological replicates). The significant of differences was assessed by Student’s *t*-test (^∗^*P* < 0.05, ^∗∗^*P* < 0.01).

Mitochondria serve as the sites of oxygen consumption as well as one of the sources of cellular ROS (Salvato et al., 2014). The results of the present study indicated that inhibition of mtETC limited the electron transfer process, which in turn facilitated electron leakage from the mtETC that results in the release of more ROS. Therefore, we evaluated the ROS in anthers with a cytochemical assay of the reaction of hydrogen peroxide using cerium chloride (**Figure 5A**). At the binucleate stage, we detected high levels of hydrogen peroxide at the mitochondrial outer membranes of the pollen grains of CHA-SQ-1-treated plants but not in the control plants, indicating that increased ROS production happened in the mitochondria of CHA-SQ-1-treated plants.

In mitochondria, MnSOD plays a major role in the detoxification of mitochondrial ROS (Chen et al., 2010). Therefore, we analyzed the MnSOD expression pattern (**Figure 5B**) and its relative enzyme activity (**Figure 5C**). The expression of MnSOD in control plants was highest at the trinucleate stage; however, similar expression was found at all stages in CHA-SQ-1-treated plants. Compared with control plants, the expression level of MnSOD was significantly changed in CHA-SQ-1-treated plants from the early uninucleate to the trinucleate stage, which was repressed by 1.8-fold at the early uninucleate stage, 1.5-fold at the later-uninucleate stage, 1.9-fold at the binucleate stage, and 2.5-fold at the trinucleate stage, respectively (**Figure 5B**). Its activity was decreased in CHA-SQ-1-treated plants from the early uninucleate to the trinucleate stage (**Figure 5C**).

These results suggest that ROS generated by mtETC dysfunction could induce oxidative stress in the mitochondria of CHA-SQ-1-treated plants. MnSOD defects further promote excessive ROS release to the cytosol.

### Excessive ROS Levels and Oxidative Stress in the Anther

**FIGURE 6:**
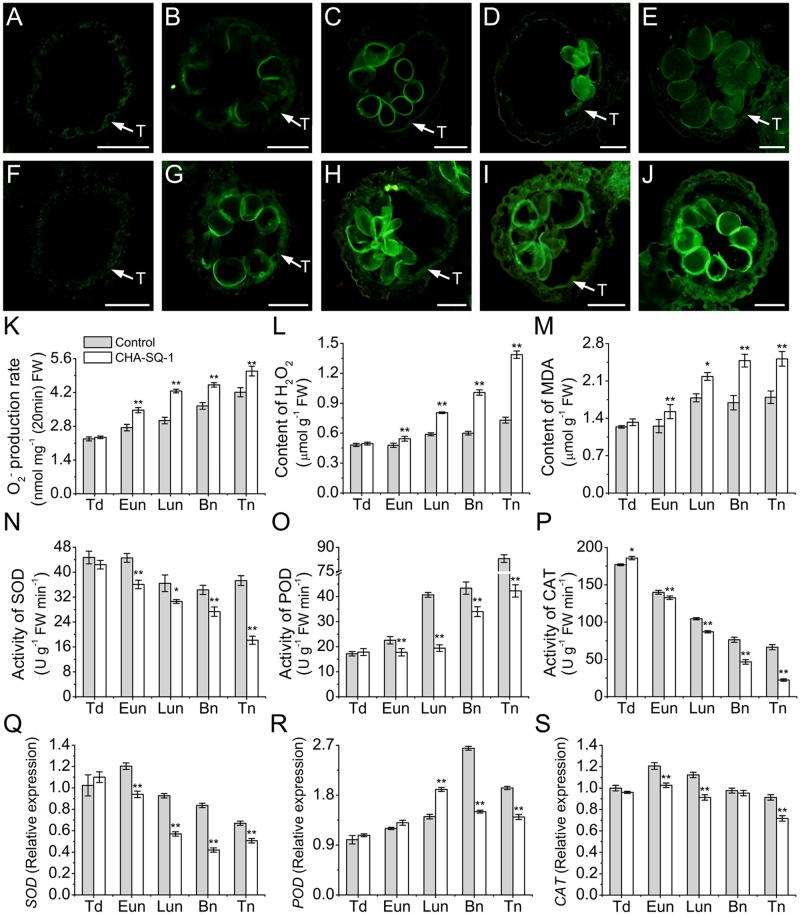
Accumulation of ROS in the anthers of CHA-SQ-1-treated wheat plants compared with its counterpart stages of control treatment. **(A–J)** Anther sections exposed to 2′,7′-dichlorodihydrofluorescein diacetate (H_2_DCF-DA) to detect ROS in the control **(A–E)** and CHA-SQ-1-treated **(F–J)** wheat plants during the tetrad stage **(A,F)**, early uninucleate stage **(B,G)**, later-uninucleate stage **(C,H)**, binucleate stage **(D,I)** and trinucleate stage **(E,J)**, respectively, using fluorescence microscope (excitation wavelength 450–490 nm). ROS is depicted by green fluorescence emission. Bars = 50 μm. **(K–P)** The O_2_^-^ production rate **(K)**, H_2_O_2_ **(L)** and MDA **(M)** levels and the activities of SOD **(N)**, POD **(O)**, and CAT **(P)** during anther development. **(Q–S)** qPCR expression levels of SOD **(Q)**, POD **(R)**, and CAT **(S)** in the anthers. Td, tetrad stage; Eun, early uninucleate stage; Lun, later-uninucleate stage; Bn, binucleate stage; Tn, trinucleate stage. Data are means ± SD of three independent experiments (biological replicates). The significant of differences was assessed by Student’s *t*-test (^∗^*P* < 0.05, ^∗∗^*P* < 0.01).

To further investigate the distribution and dynamic changes of ROS during anther development, ROS production was analyzed in anthers using the oxidant-sensitive H_2_DCF-DA (**Figures 6A–J** and **Supplementary Figure S4**). At the tetrad stage, a similar ROS accumulation and distribution pattern between control and CHA-SQ-1-treated plants was observed (**Figures 6A,F** and **Supplementary Figures S4A,F**). At the early uninucleate stage, the mtETC was inhibited within the anther of CHA-SQ-1-treated plants, along with increased ROS generation and release. Thus, the tapetum cells in CHA-SQ-1-treated plants accumulated high amounts of ROS (**Figures 6B,G**). However, there were no distinct differences in microspores at this stage (**Supplementary Figures S4B,G**). At the later-uninucleate stage, a strong fluorescent signal was observed in the tapetum cells as well as the microspores of CHA-SQ-1-treated plants (**Figures 6C,H** and **Supplementary Figures S4C,H**). Subsequently, higher intensity fluorescent signals were still detected in the tapetum cells and microspores of CHA-SQ-1-treated plants at the binucleate stage (**Figures 6D,I** and **Supplementary Figures S4D,I**) and the trinucleate stage (**Figures 6E,J** and **Supplementary Figures S4E,J**), and spread quickly to the external cell layers of the anther (epidermis and endothecium; **Figures 6I,J**). ROS concentrations was further measured (**Figures 6K–M**). The CHA-SQ-1-treated anthers showed a significantly higher O_2_^-^ level than those of the control anthers from the early uninucleate to the trinucleate stage (**Figure 6K**). It rapidly increased and consistently stayed 20% higher in CHA-SQ-1-treated anthers. Meanwhile, excess O_2_^-^ was catalyzed to form H_2_O_2_. The change of H_2_O_2_ level was the same as that of O_2_^-^, wherein an apparent increased in anther development in CHA-SQ-1-treated anthers was observed and reached a maximum value of around 190% at the trinucleate stage (**Figure 6L**). Subsequently, excess ROS induced the degradation of polyunsaturated lipids, thus forming MDA, and its level was significantly increased in the anthers of CHA-SQ-1-treated plants (maximum value: 147%; **Figure 6M**). These observations were indicative of the accumulation of excessive amount of ROS in CHA-SQ-1-treated anther.

Reactive oxygen species scavenging depends on anti-oxidative enzymes, which include SOD, CAT and POD. These enzyme activities and expression patterns were then analyzed using spectrophotometric assays (**Figures 6N–P**) and qPCR (**Figures 6Q–S**). SOD activity was significantly decreased by 19.1–105.5% in CHA-SQ-1-treated anthers from the early uninucleate to the trinucleate stage (**Figure 6N**). In addition, its gene expression level slightly decreased by 1.3- to 2.0-fold (**Figure 6Q**). POD activity continuously increased during the anther development (**Figure 6O**). However, activity of POD remained low (26.9–109.4%) in CHA-SQ-1-treated anthers from the early uninucleate to the trinucleate stage than that in control anthers at the same developmental stages (**Figure 6O**), whereas its gene expression level significantly increased (1.3-fold) at the later-uninucleate stage, then dramatically repressed (1.8- and 1.4-fold) at the later stages of CHA-SQ-1-treated anthers (**Figure 6R**), which is a possible defensive mechanism for coping with the overproduction of ROS at these stages. In contrast, the activity of CAT, which also detoxifies hydrogen peroxide, continuously decreased during anther development (**Figure 6P**). Also, CAT activity was lower (5.5–196.6%) in CHA-SQ-1-treated anthers from the early uninucleate to the trinucleate stage than that in the control anthers (**Figure 6P**). Similarly, its transcript was significantly repressed by 1.2-fold at the early- and later-uninucleate stages, and 1.3-fold at the trinucleate stage compared to the corresponding stages of control anthers (**Figure 6S**). Therefore, anti-oxidative enzymes were significantly diminished in CHA-SQ-1-treated anthers during excess ROS production, and this further disrupted oxidative/anti-oxidative homeostasis.

These results indicated that the ROS production overwhelmed its antioxidant capacity, which disrupted the balance between ROS production and release in CHA-SQ-1-treated anthers. These conditions led to the occurrence of severe oxidative stress during pollen development.

### *Type II Metacaspase* in Wheat (*TaMCAII*) Related to Abnormal Anther Apoptosis

Excessive ROS *in vivo* may generate intermediate signals that are involved in apoptosis (Maxwell et al., 2002). Meanwhile, we previously reported that the CHA-SQ-1-treated plants undergo premature tapetal PCD (Wang et al., 2015a), and affected the degradation of the cells of the tapetum and the ROS levels showed a time-dependent increase.

Recent studies have shown that *TaMCAII* plays a regulatory role during apoptosis in plants (Dudkiewicz and Piszczek, 2012; Nobili et al., 2014). Phylogenetic analyses revealed that *TaMCAII* is the closest relative of *Oryza sativa* and *Zea mays* type II metacaspase (**Supplementary Figure S5**). To test whether *TaMCAII* is involved in apoptosis of CHA-SQ-1-treated anthers, qPCR was used to analyze the expression levels (**Supplementary Figure S6**). The *TaMCAII* gene showed a significantly increase at the early uninucleate stage (1.1-fold), later-uninucleate stage (1.9-fold), and trinucleate stage (2.1-fold) in the treated plant anthers. The results clearly indicated that *TaMCAII* was involved in abnormal apoptosis of premature tapetal degradation in CHA-SQ-1-treated plants. In addition, apoptosis and the simultaneous overexpression of *TaMCAII* were also observed in controls. The binucleate stage and the trinucleate stage, which are closer to anther dehiscence, have entered into the apoptosis process and prepared for anther dehiscence. This observation further confirmed that *TaMCAII* participated in the apoptosis of anther dehiscence.

Taken together, these findings indicate that chronic oxidative stress accompanied by overexpression of *TaMCAII* triggers the abnormal apoptosis and consequently results in microspore abortion.

### Microspore FDA Viability Assay

Cells that have an intact cell membrane and respiratory activity are stained fluorescently green by FDA, and non-FDA-fluorescing cells are considered to be dead (Singh et al., 2015a). To further elucidate microspore viability during pollen abortion, microspores were stained with FDA (**Supplementary Figure S7**). At the tetrad stage, the control and CHA-SQ-1-treated plant microspores showed distinct FDA signals (**Supplementary Figure S7A**), with high vitality (98% survival; **Supplementary Figure S7B**). However, the FDA signal appeared weaker in treated plant microspores from the early uninucleate stage (91.6% survival) and reached a minimum level at the trinucleate stage (29.5% survival) in comparison with the control microspores (94.5% at the early uninucleate stage, 93.4% at the later-uninucleate stage, 97.4% at the binucleate stage and 98.2% at the trinucleate stage). These dynamic changes were consistent with the observed excessive ROS levels (**Supplementary Figures S4**, **S7**). These results implied that disrupted cell membranes and inhibited respiratory activity caused by excessive ROS results in increased membrane permeability, metabolic disturbances, and eventually visible pollen with nil or low-viability.

## Discussion

### CHA-SQ-1 Treated Induced Male-Sterile Wheat

Wheat is a self-pollinating crop that has a closed floret. In the present study, CHA-SQ-1 is rapidly absorbed by wheat, and can induce complete (100%) male sterility, which modifies its reproductive biology thus ensuring cross-pollination in cleistogamous wheat flowers. The opening of wheat flowers lasted longer than one week and was more than adequate for cross-pollination to take place. Meanwhile, the peak period for stigma receptivity lasted for 4–5 days, allowing flexibility for hybrid seed generation. CHA-SQ-1 facilitates hybrid seed production without the risk of affecting important agronomic traits and thus has great potential for the development of commercial wheat hybrids (Song et al., 2014; Wang et al., 2015a). More importantly, CHA-induced male sterility, with exact the same nuclear background, may circumvent the confounding factors of genotype in CMS and genic male sterility (Cheng et al., 2013; Song et al., 2015). This provides a shortcut for revealing the mechanism of male sterility.

### Mitochondrial Dysfunction in the CHA-SQ-1-Treated Plants

The plant trait of CMS is often determined by mitochondrial dysfunction and is characterized by a sterile pollen phenotype (Wang K. et al., 2013). However, to date, the relationship between mitochondria and CHA-induced male sterility in wheat has not been established. In these results, mitochondria was proved to be potential targets for CHA-SQ-1, which have been shown to be efficiently transported from leaves to flowers in wheat (Zhu et al., 2015). Recent studies on plant mitochondria indicate that inhibited mtETC reduces pollen grain production and/or causes sterility (Wang K. et al., 2013; Chen and Liu, 2014). Similarly, COX activity and its protein level are significantly reduced in the CHA-SQ-1-treated anthers, which indicate that COP of electron transport is inhibited. These results are consistent with a possible leakage of electrons from impaired ETC that reduces molecular O_2_ to superoxide and H_2_O_2_. Meanwhile, CHA-SQ-1 also increases electron flow via the AOP, which was confirmed by the overexpression of *AOX* genes and the increased AOP activity. This may be a broader role for the AOP and one way of protecting against extreme conditions; although the increase in the capacity of AOP limits the production of O_2_ free radicals under stress conditions, it was clearly not sufficient to restore electron flow to the normal COP. The decline in mitochondrial respiration rates is obviously a result of damage to the more sensitive COP. Moreover, mitochondrial membrane ATPase (F_1_F_0_ ATP synthase or complex V) plays a critical role in energy metabolism, mainly by converting ADP into ATP via a transmembrane proton gradient (Sabar et al., 2003), and thus serves as an indicator of mitochondrial activity. Our findings showed that the ATPase activity and its protein level in the CHA-SQ-1-treated anthers were distinctly lower than that in the control, which indicated that reduced ATP levels caused by inhibited mtETC could limit the access to ATP that could be utilized during pollen development. Maize tapetal and pollen grains have roughly 40 and 20 times the levels of mitochondria that are observed in vegetative tissues (Warmke and Lee, 1978); similar results were observed in the present study (**Supplementary Figure S8**). These results were in agreement with high ATP utilization during anther development. The gap between the energy expenditure and mitochondrial energy-production may be caused by the disruption of mtECT, and further deteriorate the mitochondrial dysfunction and the promoting of ETC related to ROS production. This may be the physiological causes for the CHA-induced male sterility in wheat.

### Reactive Oxygen Species in Mitochondria-Mediated Apoptosis in Anthers

Despite of the presence of a highly efficient mitochondrial/cellular anti-oxidant system, the superoxide anion was still produced constantly due to the significant electron leakage during electron transfer, rendering mitochondria as the major source of endogenous ROS (Borges et al., 2014). Our observation of defective mitochondria as more efficient producers of ROS supports this conclusion. MnSOD is the major anti-oxidant defense enzyme in mitochondria, and plays an important role for cells’ primary defense against free radical-mediated damage, which is encoded in the nucleus but localized in mitochondria (Chen et al., 2010). In this study, we observed a negative correlation between ROS level and MnSOD activity, which exacerbates the sustained accumulation of ROS, thus leading to abnormally high levels of oxidative stress in the mitochondria. Then, the excess accumulation of ROS causes oxidative damage and, apart from hydroxyl radicals, destroys cell membranes and lipoproteins via a process called lipid peroxidation (determined by MDA content). Finally, excessive ROS is released to the cytosol. Meanwhile, we found that the increase of ROS content was accompanied by inhibited expression and activity of SOD, CAT and POD. Excessive ROS was not effectively eradicated by the anti-oxidative system, which made microspores suffer oxidative stress during pollen development. Then, the chronic oxidative stress triggered apoptosis and consequently, and resulted in microspore abortion.

### A Proposed Model of Mitochondria-Mediated Anther Apoptosis in CHA-SQ-1-Induced Male Sterile Wheat

Plant male sterility is of particular significance for developmental and molecular studies because of its usefulness in hybrid seed production (Eckardt, 2006). This study first reported the mechanism underlying male sterility as modulated by mitochondrial in CHA-SQ-1-induced male sterile wheat, which caused by dysfunctional pollen and disrupted anther development; vegetative development as well as female fertility followed the normal route (**Figure 1**).

**FIGURE 7:**
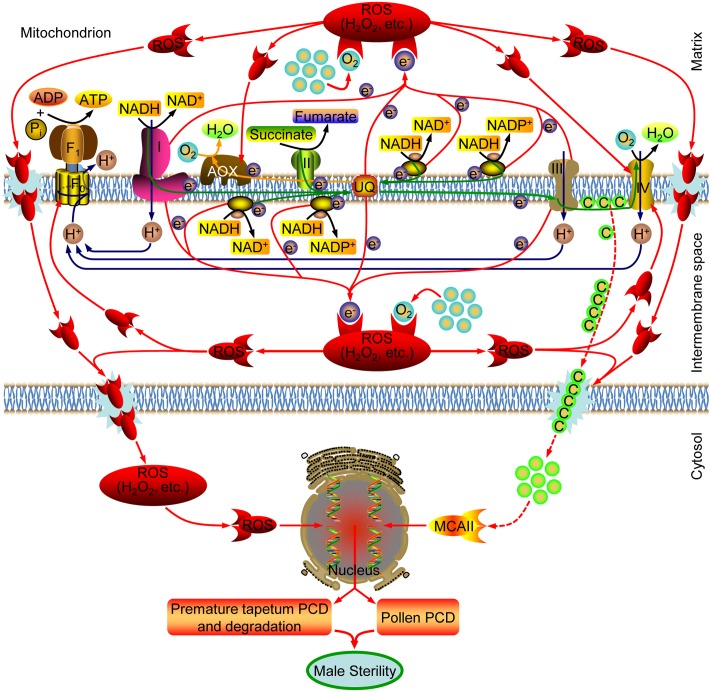
A model for the mechanism underlying CHA-SQ-1-induced male sterility in wheat. CHA-SQ-1 impairs mitochondrial function. Inhibited mtETC causes increased ROS production (H_2_O_2_, etc.), oxidative burst, and then result in membrane disruption, permeability increase and possible cytochrome *c* (Cyt *c*) release, which directly or indirectly leads to premature tapetal degeneration, microspore programmed cell death (PCD) and ultimately induces male sterility in wheat.

Based on previous reports and findings, a tentative model of the molecular mechanism of CHA-SQ-1-induced male sterility in wheat was proposed, as summarized in **Figure 7**. CHA-SQ-1 impairs mitochondrial function by depressing the levels of COX and ATP protein, inhibiting the activity of COP. The COP (the primary electron transfer pathway) is inhibited by dysfunctional mtETC in CHA-SQ-1-treated plant anthers. Some electrons fail to pass through the COP to combine with oxygen and produce water, causing ubiquinone to reach an extremely low level. Recent plant mitochondrial studies indicate that mtETC complexes I and II serve as sites for ROS (Chen et al., 2003; Rhoads et al., 2006), and NADPH dehydrogenase is a potential site for ROS (Moller, 2001). Thus, excess electrons directly combine with molecular oxygen in the immediate vicinity via these complexes (complexes I and III, NADPH dehydrogenase and the high reduction level of ubiquinone), instead of the next carrier in the chain, to form ROS. A recent study has discovered that the activation state of AOX is regulated by the redox state of the enzyme and the intracellular pyruvate concentration (van Dongen et al., 2011). In this work, increased AOX expression and AOP activity captured some excess electrons but did not reduce the level of ROS in the mitochondria. Then, the AOP of electron transport in the mtETC was also inhibited by the excessive amounts ROS. Excessive ROS accelerate peroxidation of membrane lipids, and result in membrane disruption, permeability increasing and metabolic disturbances, combined with MnSOD defects in the mitochondria of CHA-SQ-1-treated plant anthers, large amounts of ROS and cyt *c* are released into the cytoplasm (Luo et al., 2013). The reduction of cyt *c* in the intermembranous space not only further inhibits the COP of mtETC, but also accelerates ROS production, which creates a vicious cycle in the mitochondria of CHA-SQ-1-treated plant anthers. Meanwhile, we have also observed that the number of mitochondria was higher in microspores and the tapetum than that in any other tissues, particularly in the tapetum (**Supplementary Figure S8**). This is one of the reasons that the fluorescent signal of ROS and apoptosis were first detected in tapetum at the early uninucleate stage, then in microspore at the later-uninucleate stage. *TaMCAII*, an apoptotic factor, has been shown to be implicated in apoptosis of wheat tissues (Dudkiewicz and Piszczek, 2012; Nobili et al., 2014). Although evidence for apoptosome formation has not been established in plants, release of cyt *c* has been extensively studied in plant apoptosis (Balk and Leaver, 2001; Luo et al., 2013), combined with the enhanced expression of *TaMCAII* in CHA-SQ-1-treated plant anthers, it is inferred that *TaMCAII* might be activated by the released cyt *c* that is involved in apoptosis. Studies have also suggested that ROS is able to induce apoptosis (Fleury et al., 2002; Darehshouri et al., 2008; He et al., 2008; Li et al., 2012; Zhao et al., 2015), and apoptosis is a “ready-to-be-activated” process in tapetum cells (Navrot et al., 2007). TUNEL analysis provided direct evidence that abnormal apoptosis started at the early uninucleate stage in the tapetum and the later-uninucleate stage in the microspore. In addition, ROS and apoptosis also affect other regions of the anther and the vascular bundle cells that block their own nutrient biosynthesis, foreign substances importation and energy metabolism, i.e., ATP, sucrose (**Supplementary Figure S9A**) and starch (**Supplementary Figure S9B**). Microspore viability was significantly weakened and pollen abortion eventually occurred.

## Author Contributions

SW and GZ designed the study and wrote the manuscript. SW, YiZ, QS, ZF, ZC, YaZ, and LilZ participated in experiments. LinZ, NN, SM, JW, YY, and ZH, discussed the results and revised the manuscript. All authors have read and approved the final manuscript.

## Conflict of Interest Statement

The authors declare that the research was conducted in the absence of any commercial or financial relationships that could be construed as a potential conflict of interest. The reviewer SH declared a shared affiliation, with no collaboration, with several of the authors, QS, ZC, YaZ, LilZ, NN, SM, JW, YY, GZ, to the handling Editor.

## Supplementary Material

FIGURE S1 Proteome profiles of floret mitochondria in the control **(A,B)** and the CHA-SQ-1-treated **(C,D)** wheat plants at the early uninucleate **(A,C)** and trinucleate **(B,D)** stages. Mitochondrial proteins (160 μg) were loaded on IPG gel strips (17-cm; pH 4–7), then followed by SDS-PAGE on a vertical slab gel (11%). Proteins were visualized using silver staining. Numbered spots represent the identifications detailed in **Supplementary Table S2**.

FIGURE S2 DAPI-stained showing microspore development. Five stages of microspore development in control **(A–E)** and the corresponding stages of the CHA-SQ-1-treated plants **(F–J)** were compared. **(A,F)** tetrad stage. **(B,G)** Early uninucleate stage. **(C,H)** Later-uninucleate stage. **(D,I)** Binucleate stage. **(E,J)** Trinucleate stage. Bars = 10 μm.

FIGURE S3 Multiple alignments of *COX2* protein sequences of eight plant species. Accession numbers of the protein sequences are given in parenthesis: TaCOX2 of *Triticum aestivum* (COX2 WHEAT), OsCOX2 of *Oryza sativa* (CAA25566), SbCOX2 of *Sorghum bicolor* (YP 762349), ZmCOX2 of *Zea mays* (COX2 MAIZE), AtCOX2 of *Arabidopsis thaliana* (NP 085487), GmCOX2 of *Glycine max* (COX2 SOYBN), BrCOX2 of *Brassica rapa* subsp. *oleifera* (AAB92666), and StCOX2 of *Solanum tuberosum* (ABB43241). The sequence alignment was performed using the BioEdit software.

FIGURE S4 Analysis of dynamic changes of ROS in microspores. Microspores treated with H_2_DCF-DA to detect ROS in control **(A–E)** and CHA-SQ-1-treated **(F–J)** wheat plants at five stages. Presence of ROS is indicated by green fluorescent signals. **(A,F)** tetrad stage; **(B,G)** early uninucleate stage; **(C,H)** later-uninucleate stage; **(D,I)** binucleate stage; **(E,J)** trinucleate stage. Bars = 50 μm.

FIGURE S5 Maximum likelihood phylogenetic tree obtained for the alignment of peptide sequences of the type II metacaspase clan. The alignments were generated using MEGA v. 6.06 and tree branches were bootstrapped with 1,000 replications. The solid black circle indicates the sequences used in the present study.

FIGURE S6 qPCR assays for the analysis of *TaMCAII* expression levels in anthers at different developmental stages. Td, tetrad stage; Eun, early uninucleate stage; Lun, later-uninucleate stage; Bn, binucleate stage; Tn, trinucleate stage. Data are means ± SD of three independent experiments (biological replicates). The significant of differences was assessed by Student’s *t*-test (^∗^*P* < 0.05, ^∗∗^*P* < 0.01).

FIGURE S7 Analysis of survival rate of microspores in control and CHA-SQ-1-treated wheat plants. **(A)** Microspores were stained using fluorescein diacetate (FDA) to determine viability and membrane integrity of control and CHA-SQ-1-treated wheat plants at the five different stages, as viewed using fluorescence microscope (excitation wavelength: 450–490 nm). FDA signals are green in color, whereas microspores with relatively weak to no signals were described as low-viability or dead (see white *arrowhead*). Bars = 50 μm. **(B)** The percentages of normal microspores at each stage in anthers of control plants or plants treated with CHA-SQ-1. Td, tetrad stage; Eun, early uninucleate stage; Lun, later-uninucleate stage; Bn, binucleate stage; Tn, trinucleate stage. Data are means ± SD of three independent experiments (biological replicates). The significant of differences was assessed by Student’s *t*-test (^∗^*P* < 0.05, ^∗∗^*P* < 0.01).

FIGURE S8 The distribution of mitochondria in anther by transmission electron microscopy. Compared to other layers of the anther **(A)**, the density of mitochondria (some of them are indicated by black *arrowheads*) is higher in the tapetum **(B)** and microspores **(C)**. E, epidermis; En, endothecium; ML, middle layer; T, tapetum; P, pollen grain wall; N, nucleus. Bars = 5 μm **(A,C)**, 0.5 μm **(B)**.

FIGURE S9 Soluble sugar **(A)** and starch **(B)** levels in anther. Td, tetrad stage; Eun, early uninucleate stage; Lun, later-uninucleate stage; Bn, binucleate stage; Tn, trinucleate stage. Data are means ± SD of three independent experiments (biological replicates). The significant of differences was assessed by Student’s *t*-test (^∗^*P* < 0.05, ^∗∗^*P* < 0.01).

TABLE S1 Gene-specific primers used in this study.

TABLE S2 Identification of six differentially expressed proteins from isolated mitochondria of control and CHA-SQ-1-treated wheat plants.
