# Supplementary figures and images for: Mitochondrial Dysfunction Causes Oxidative Stress and Tapetal Apoptosis in Chemical Hybridization Reagent-Induced Male Sterility in Wheat
Source: Front Plant Sci. 2018 Jan 10;8:2217. doi: 10.3389/fpls.2017.02217 (PMC5767846; doi:10.3389/fpls.2017.02217)

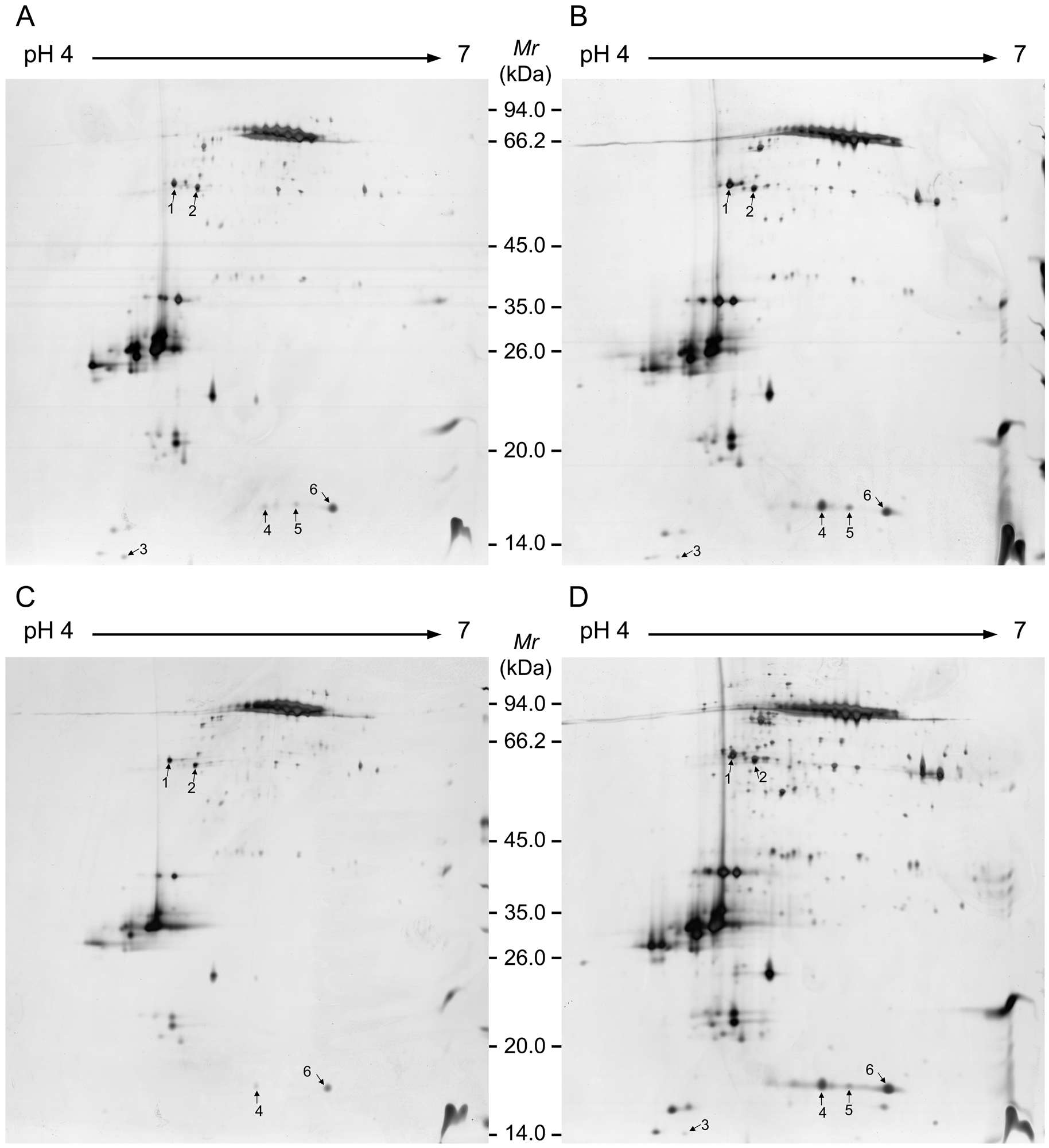

Supplement: FIGURE S1 — Proteome profiles of floret mitochondria in the control (A,B) and the CHA-SQ-1-treated (C,D) wheat plants at the early uninucleate (A,C) and trinucleate (B,D) stages. Mitochondrial proteins (160 μg) were loaded on IPG gel strips (17-cm; pH 4–7), then followed by SDS-PAGE on a vertical slab gel (11%). Proteins were visualized using silver staining. Numbered spots represent the identifications detailed in Supplementary Table S2. [file Image_1.TIF]

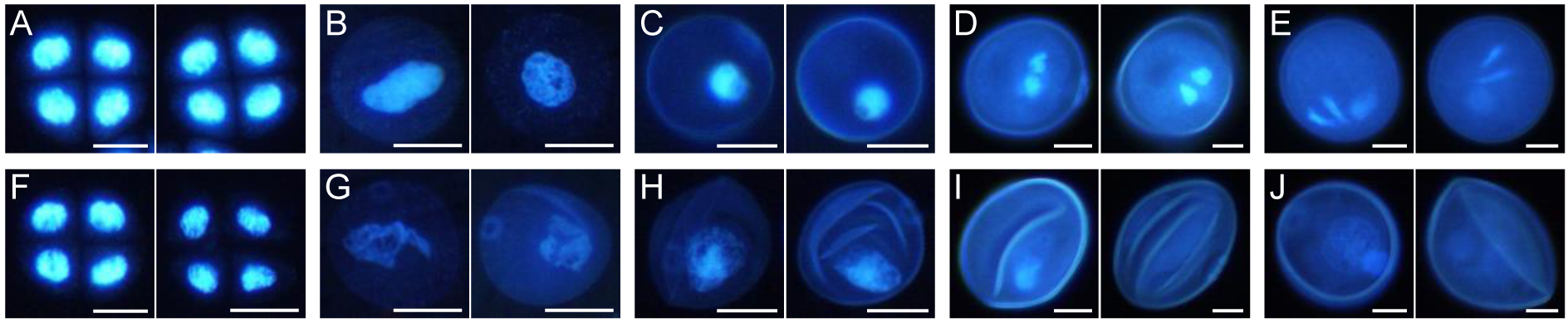

Supplement: FIGURE S2 — DAPI-stained showing microspore development. Five stages of microspore development in control (A–E) and the corresponding stages of the CHA-SQ-1-treated plants (F–J) were compared. (A,F) tetrad stage. (B,G) Early uninucleate stage. (C,H) Later-uninucleate stage. (D,I) Binucleate stage. (E,J) Trinucleate stage. Bars = 10 μm. [file Image_2.TIF]

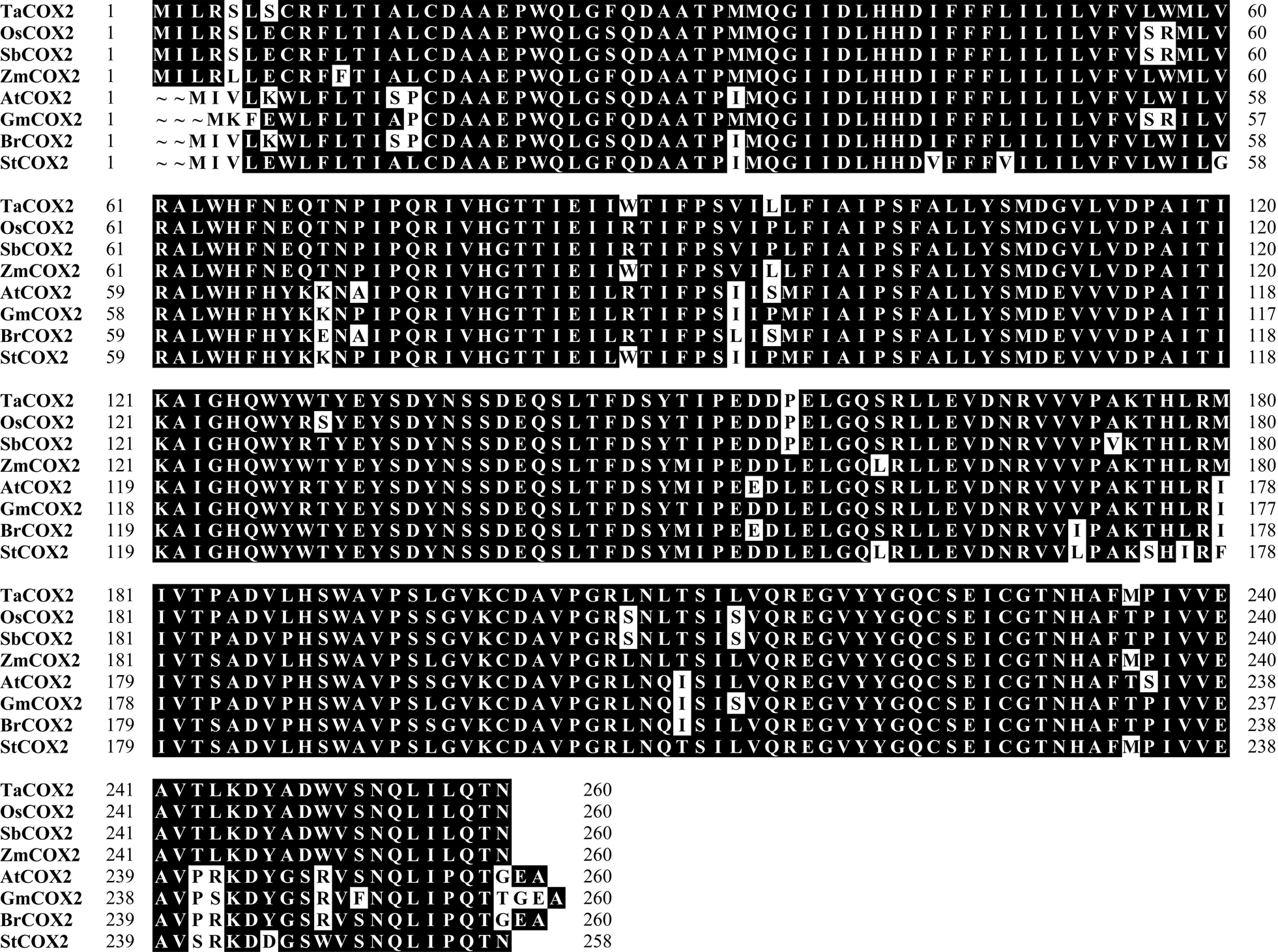

Supplement: FIGURE S3 — Multiple alignments of COX2 protein sequences of eight plant species. Accession numbers of the protein sequences are given in parenthesis: TaCOX2 of Triticum aestivum (COX2 WHEAT), OsCOX2 of Oryza sativa (CAA25566), SbCOX2 of Sorghum bicolor (YP 762349), ZmCOX2 of Zea mays (COX2 MAIZE), AtCOX2 of Arabidopsis thaliana (NP 085487), GmCOX2 of Glycine max (COX2 SOYBN), BrCOX2 of Brassica rapa subsp. oleifera (AAB92666), and StCOX2 of Solanum tuberosum (ABB43241). The sequence alignment was performed using the BioEdit software. [file Image_3.TIF]

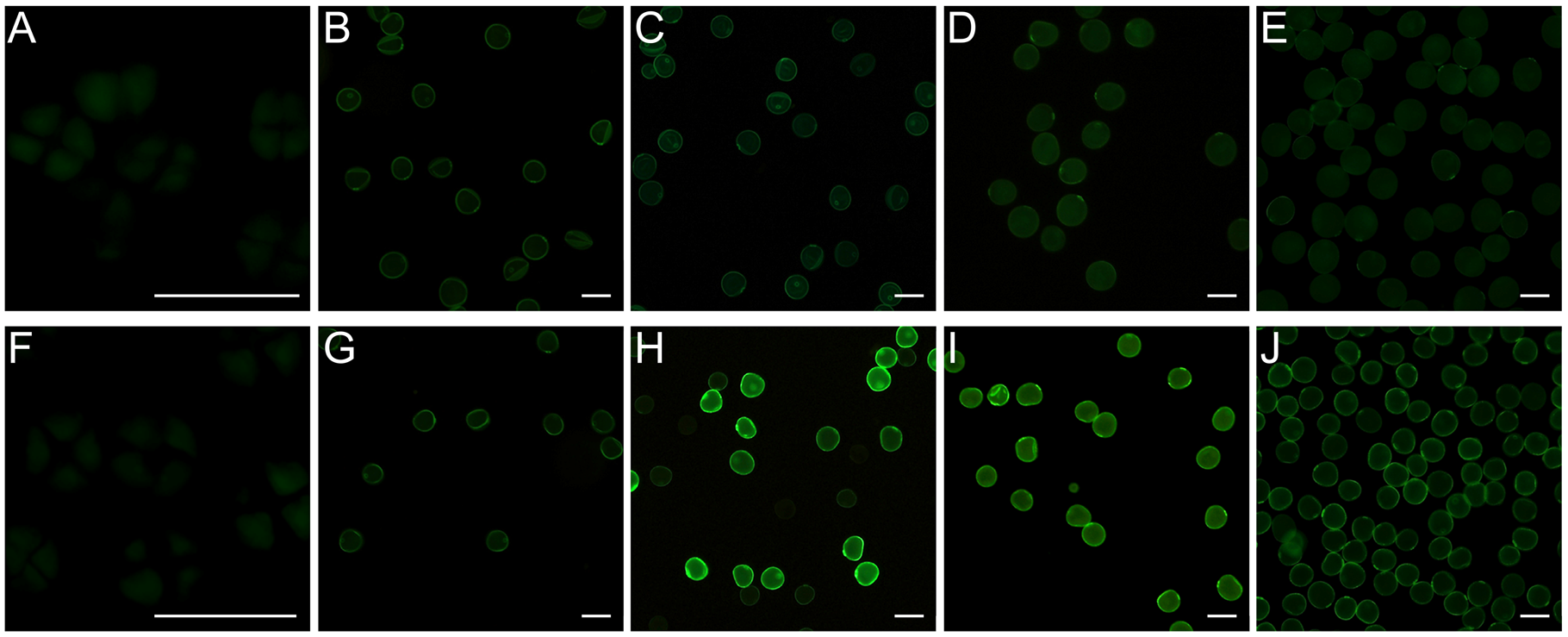

Supplement: FIGURE S4 — Analysis of dynamic changes of ROS in microspores. Microspores treated with H2DCF-DA to detect ROS in control (A–E) and CHA-SQ-1-treated (F–J) wheat plants at five stages. Presence of ROS is indicated by green fluorescent signals. (A,F) tetrad stage; (B,G) early uninucleate stage; (C,H) later-uninucleate stage; (D,I) binucleate stage; (E,J) trinucleate stage. Bars = 50 μm. [file Image_4.TIF]

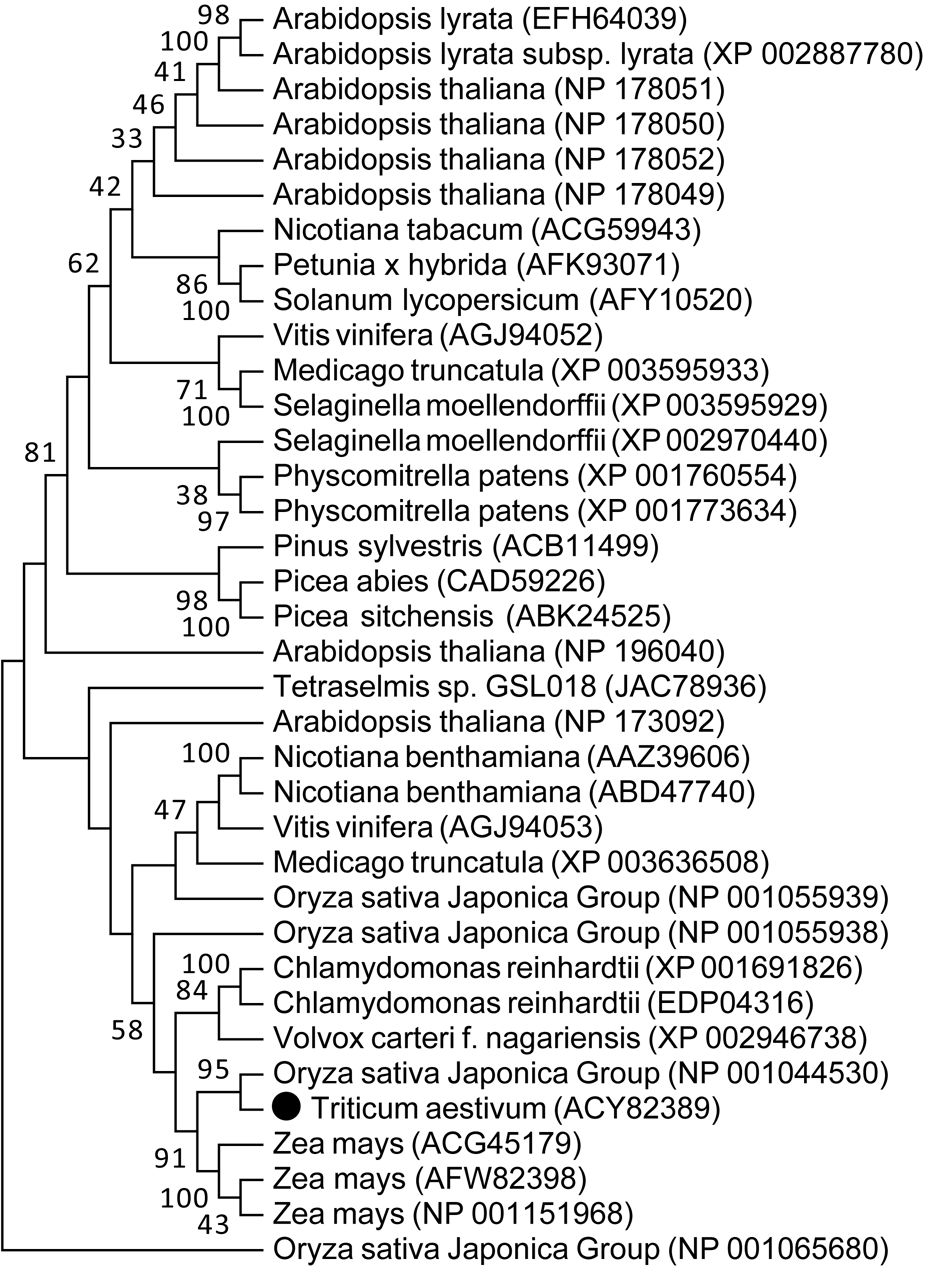

Supplement: FIGURE S5 — Maximum likelihood phylogenetic tree obtained for the alignment of peptide sequences of the type II metacaspase clan. The alignments were generated using MEGA v. 6.06 and tree branches were bootstrapped with 1,000 replications. The solid black circle indicates the sequences used in the present study. [file Image_5.TIF]

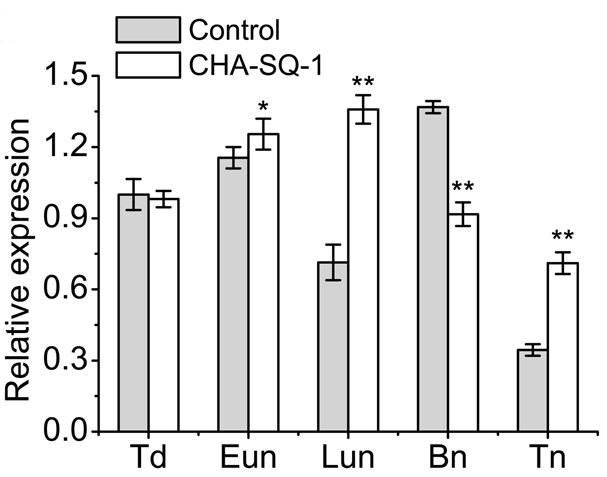

Supplement: FIGURE S6 — qPCR assays for the analysis of TaMCAII expression levels in anthers at different developmental stages. Td, tetrad stage; Eun, early uninucleate stage; Lun, later-uninucleate stage; Bn, binucleate stage; Tn, trinucleate stage. Data are means ± SD of three independent experiments (biological replicates). The significant of differences was assessed by Student’s t-test (∗P < 0.05, ∗∗P < 0.01). [file Image_6.TIF]

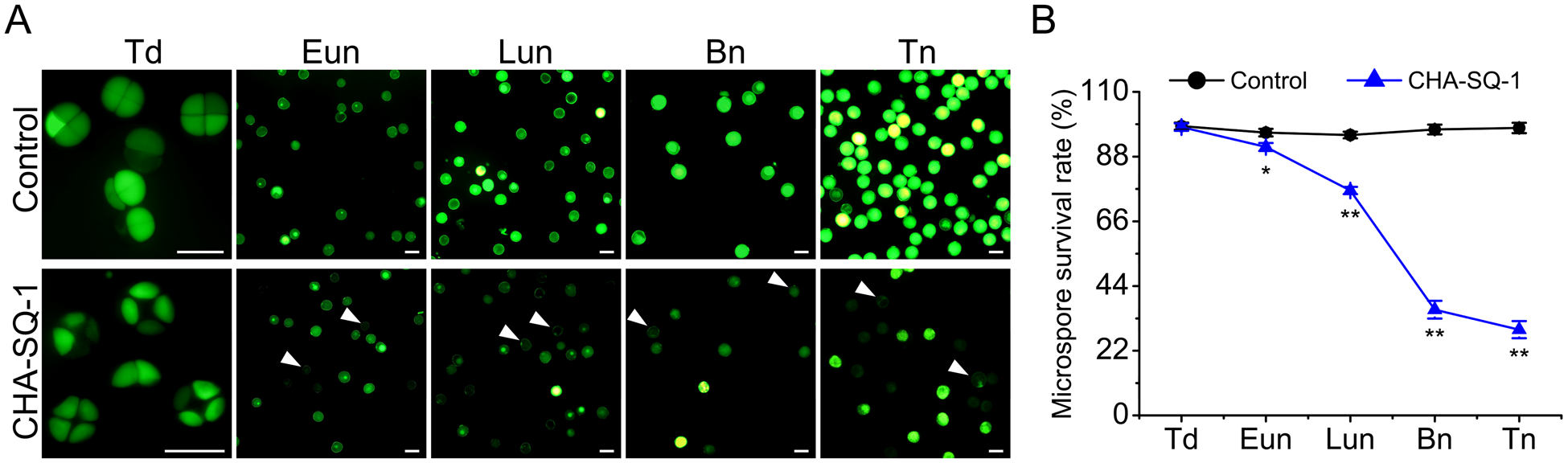

Supplement: FIGURE S7 — Analysis of survival rate of microspores in control and CHA-SQ-1-treated wheat plants. (A) Microspores were stained using fluorescein diacetate (FDA) to determine viability and membrane integrity of control and CHA-SQ-1-treated wheat plants at the five different stages, as viewed using fluorescence microscope (excitation wavelength: 450–490 nm). FDA signals are green in color, whereas microspores with relatively weak to no signals were described as low-viability or dead (see white arrowhead). Bars = 50 μm. (B) The percentages of normal microspores at each stage in anthers of control plants or plants treated with CHA-SQ-1. Td, tetrad stage; Eun, early uninucleate stage; Lun, later-uninucleate stage; Bn, binucleate stage; Tn, trinucleate stage. Data are means ± SD of three independent experiments (biological replicates). The significant of differences was assessed by Student’s t-test (∗P < 0.05, ∗∗P < 0.01). [file Image_7.TIF]

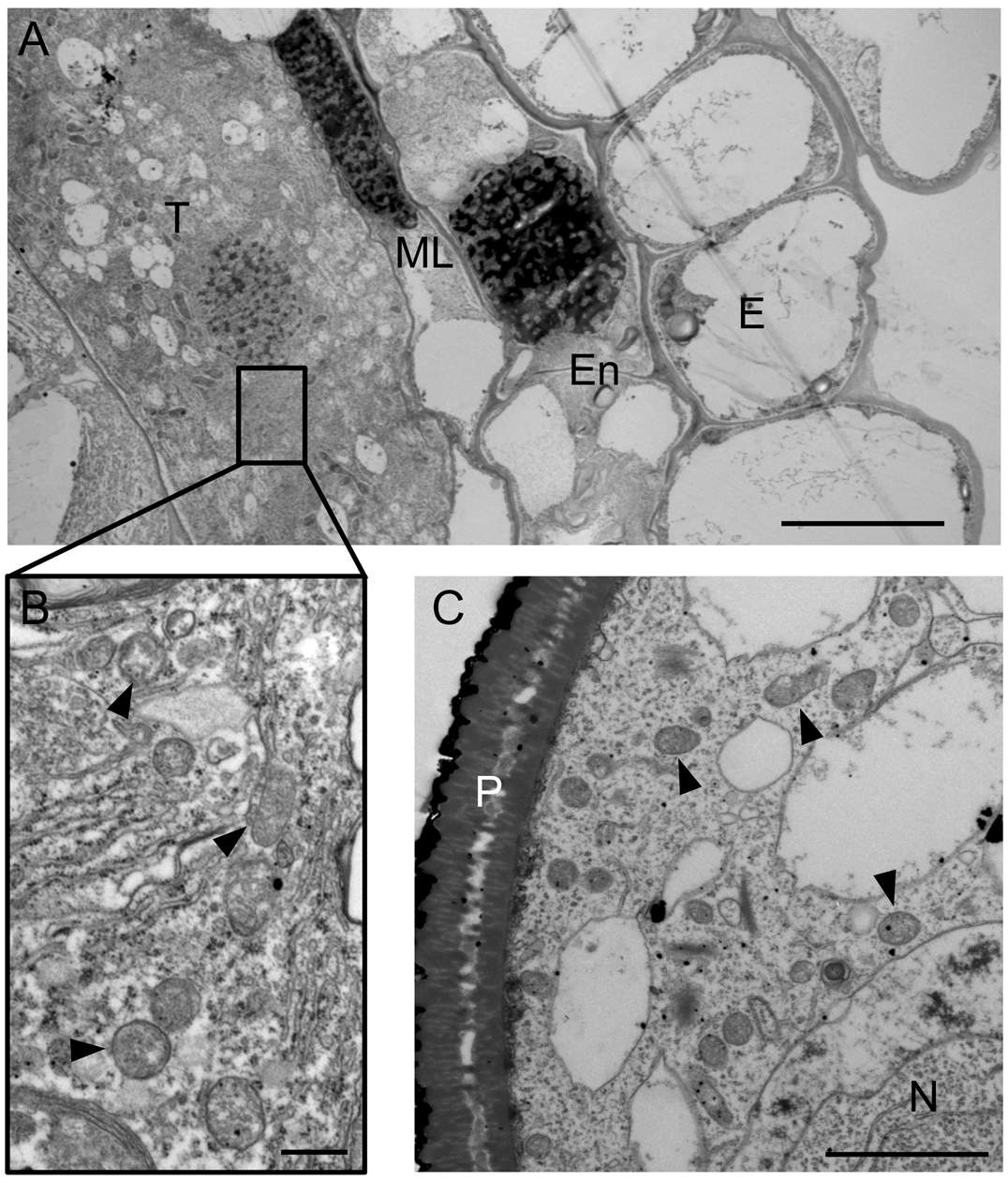

Supplement: FIGURE S8 — The distribution of mitochondria in anther by transmission electron microscopy. Compared to other layers of the anther (A), the density of mitochondria (some of them are indicated by black arrowheads) is higher in the tapetum (B) and microspores (C). E, epidermis; En, endothecium; ML, middle layer; T, tapetum; P, pollen grain wall; N, nucleus. Bars = 5 μm (A,C), 0.5 μm (B). [file Image_8.TIF]

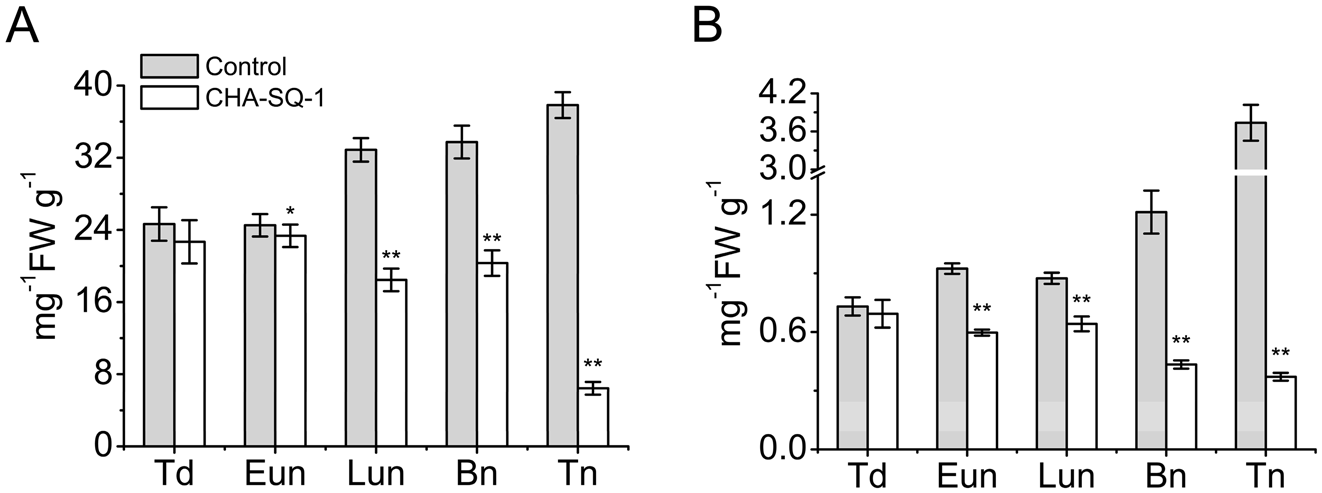

Supplement: FIGURE S9 — Soluble sugar (A) and starch (B) levels in anther. Td, tetrad stage; Eun, early uninucleate stage; Lun, later-uninucleate stage; Bn, binucleate stage; Tn, trinucleate stage. Data are means ± SD of three independent experiments (biological replicates). The significant of differences was assessed by Student’s t-test (∗P < 0.05, ∗∗P < 0.01). [file Image_9.TIF]
